# *PIK3CA* mutation testing as a valuable molecular surrogate for lipomatosis of the median nerve: clinicopathological and molecular analysis of six cases

**DOI:** 10.1007/s00428-023-03540-7

**Published:** 2023-04-17

**Authors:** Justus Osterloh, Abbas Agaimy, Frederick Fried, Robert Stoehr, Rolf Janka, Andreas Arkudas, Raymund E. Horch

**Affiliations:** 1 Department of Plastic and Hand Surgery and Laboratory for Tissue Engineering and Regenerative Medicine, University Hospital Erlangen, Friedrich-Alexander University Erlangen-Nürnberg (FAU), Erlangen, Germany; 2 Institute of Pathology, University Hospital of Erlangen, Friedrich-Alexander-University Erlangen-Nürnberg (FAU), Erlangen, Germany; 3 Melbourne Institute of Plastic Surgery, Melbourne, Australia; 4 Department of Radiology, University Hospital of Erlangen, Friedrich-Alexander-University Erlangen-Nürnberg (FAU), Erlangen, Germany

**Keywords:** Lipomatosis, Peripheral nerves, Neuropathy, Sequencing, mTOR, Everolimus

## Abstract

Lipomatosis of peripheral nerves (LPN, also known as fibrolipomatous or lipofibromatous hamartoma of peripheral nerves) is a very rare, benign, intraneural, tumorous lesion that predominantly involves the median nerve but may rarely affect any peripheral nerve. Recently, *PIK3CA* mutations have been reported in macrodactyly, a rare condition related to LPN, and in other localized lipomatous overgrowth syndromes. In this retrospective study, we report 6 cases of FPN involving the median nerve (4 of them identified among 570 patients with carpal tunnel syndrome who underwent surgical decompression at our center from 2012 to 2022 and two seen in consultation by one of the authors). All cases were diagnosed via biopsy or resection supplemented by MRI. Patients were 4 males and 2 females aged 23 to 60 years (mean 38 years). One patient with bilateral lesions had in addition extensive angiomatosis of the paravertebral region. Histological examination showed an abnormal amount of mature fatty tissue containing disordered fibrous bands, entrapping normal-looking nerve fibers with prominent perineurial and endoneurial fibrosis. Genetic analysis using snapshot assay constructed to detect hotspots mutations in *PIK3CA* revealed similar *PIK3CA* mutations (p.H1047R; c.3140A>G) in 5/6 cases (83.3%). Our study represents a further contribution to the literature on LPN and highlights the diagnostic value of *PIK3CA* mutation testing as surrogate tool in equivocal cases and in those lesions without associated macrodactyly, especially as the biopsy findings of this lesion are essentially nonspecific.

## Introduction

Lipomatosis of peripheral nerves (LPN, also known as fibrolipomatous or lipofibromatous hamartoma of peripheral nerves) is a very rare, benign, intraneural tumor consisting of abnormal amount of mature fibrofatty tissue [1, 2]. Its most common site is the median nerve at the wrist, volar forearm, or hand, whereas cases of LPN of the ulnar nerve as well as lesions originating at the lower extremity have also been reported [1]. The etiology of LPN is still uncertain, but a hamartomatous origin was hypothesized and associations with macrodactyly and congenital conditions (Klippel–Trenaunay syndrome, proteus syndrome) have been reported [3–5]. As the tumor slowly grows, patients with LPN suffer from symptoms due to compressive neuropathy (paresthesia, numbness, or functional limitation), which also represents typical symptoms of carpal tunnel syndrome. Occasionally, a palpable mass lesion can be observed. MRI-imaging of the lesion shows pathognomonic features with fusiform nerve enlargement associated with tumefactive fatty infiltration and coaxial-cable-like appearance. Histopathological analysis reveals normal-looking fibrofatty tissue lacking cytological atypia and other features of genuine adipocytic neoplasms. Diagnosis of LPN has been challenging. In our experience, several biopsied lesions (including some of our current cases) without detailed clinical findings or suggestion of LPN as a clinical diagnosis have received merely a descriptive histopathological reporting/diagnosis without mentioning LPN as a possibility. This was mainly due to the rarity of the lesion and, hence, limited familiarity of many pathologists with the entity and due to the fact that histological findings in biopsied LPN are nonspecific and hardly ever distinguishable from normal fibrofatty tissue. Notably, the literature on *PIK3CA* mutations has been largely limited to series addressing macrodactyly, and the association of *PIK3CA* mutations with LPN has not been much emphasized in the pathology literature. In this retrospective study, we present our experience with LPN of the median nerve with particular focus on *PIK3CA* mutation testing as surrogate for diagnosis.

## Material and methods

Our institutional archive was searched for cases of carpal tunnel syndrome and for LPN of the median nerve. Clinical examination, including detailed physical examination, was performed, and the clinical histories were extensively reviewed. Additionally, MRI-imaging data were carefully evaluated, and all available pathology specimens were re-reviewed. In addition, cases seen by one of the authors (AA) in the setting of histopathological consultation were included as well. The tissue specimens were fixed in formalin and processed routinely for histopathology. Hematoxylin and eosin (H&E) slides were reviewed retrospectively to exclude lipomatous neoplasms.

## Genetic analysis

For DNA extraction, the whole specimen was marked on an H&E-stained slide in 5 of the cases. In one case, the neural tissue and the fibrolipomatous component were marked for manual microdissection to test the two components separately. DNA was isolated using the Blood DNA Preparation Kit (Maxwell® 16 System; Promega Corporation) according to the manufacturer’s protocols. A previously described highly sensitive SNaPshot© multiplex assay, based on the SNaPshot Multiplex System assay (Applied Biosystems, Foster City, CA, USA), was used to screen for three mutation hotspot regions in the *PIK3CA* gene (codons 542, 545, and 1047) [3].

## Results

### Frequency of LPN between clinical carpal tunnel syndrome cohort and soft tissue consultation cases

During the period 2012 to 2022, a total of 570 patients underwent surgical decompression of the carpal tunnel at our center based on a clinical diagnosis of carpal tunnel syndrome. Among them, we identified four patients suffering from carpal tunnel syndrome caused by a compressive neuropathy of the median nerve due to LPN (0.7%). In addition to these four in-house patients, two cases were retrieved from the histopathological consultation files of one of the authors (AA). One consultation case originating in the lower limb (confirmed by *PIK3CA* mutation) was excluded to maintain homogeneity of the series.

### Clinical-demographic characteristics, imaging findings, and treatment of patient with LPN

The clinical and pathological findings of the six patients are outlined in Table 1.

**Table 1:** Clinicopathological and molecular characteristics of patients with lipomatosis of the median nerve

Patient	Age/sex	DML	Therapy	Histopathological findings/original diagnosis	PIK3CA mutation results	Other associated diseases
1	27/F	7.4 ms	Surgical decompression and biopsy	Fatty and fibrotic tissue, atypical overgrowth of vessels/descriptive reporting	p.H1047R; c.3140A>G	-
2	50/F	NA	Surgical decompression and biopsy, revision after 10 years and fascial sheet	Hamartomatous fibrolipomatous soft tissue/FLH	p.H1047R; c.3140A>G	Bilateral lipomatosis of median nerve + Paravertebral soft tissue angiomatosis
3	28/M	9.1 ms	Surgical decompression, biopsy, neurolysis of median nerve and fascial sheet	Fatty and fibrotic tissue with prominent collagen fibers/descriptive reporting	Wild-type	-
4	40/M	9.5 ms	Surgical decompression in external center, referral to our center: biopsy, neurolysis of median nerve and fascial sheet	Fibrotic tissue with numerous vessels and little neural tissue/descriptive reporting	p.H1047R; c.3140A>G	-
5	60/M	NA	Surgical decompression in external center	Fibrofatty tissue, thick-walled blood vessels, numerous nerve fascicles and perineural fibrosis/FLH	p.H1047R; c.3140A>G*	-
6	23/M	NA	Surgical decompression in external center	Fibrolipomatous soft tissue with increased thick-walled vessels and nerve fibers/FLH	p.H1047R; c.3140A>G	-

*Nerves and fat tested separately (manual microdissection) and revealed same mutation in both components. *DML*, distal motor latency; *FLH*, fibrolipomatous hamartoma; *NA*, not available

**Fig. 1:**
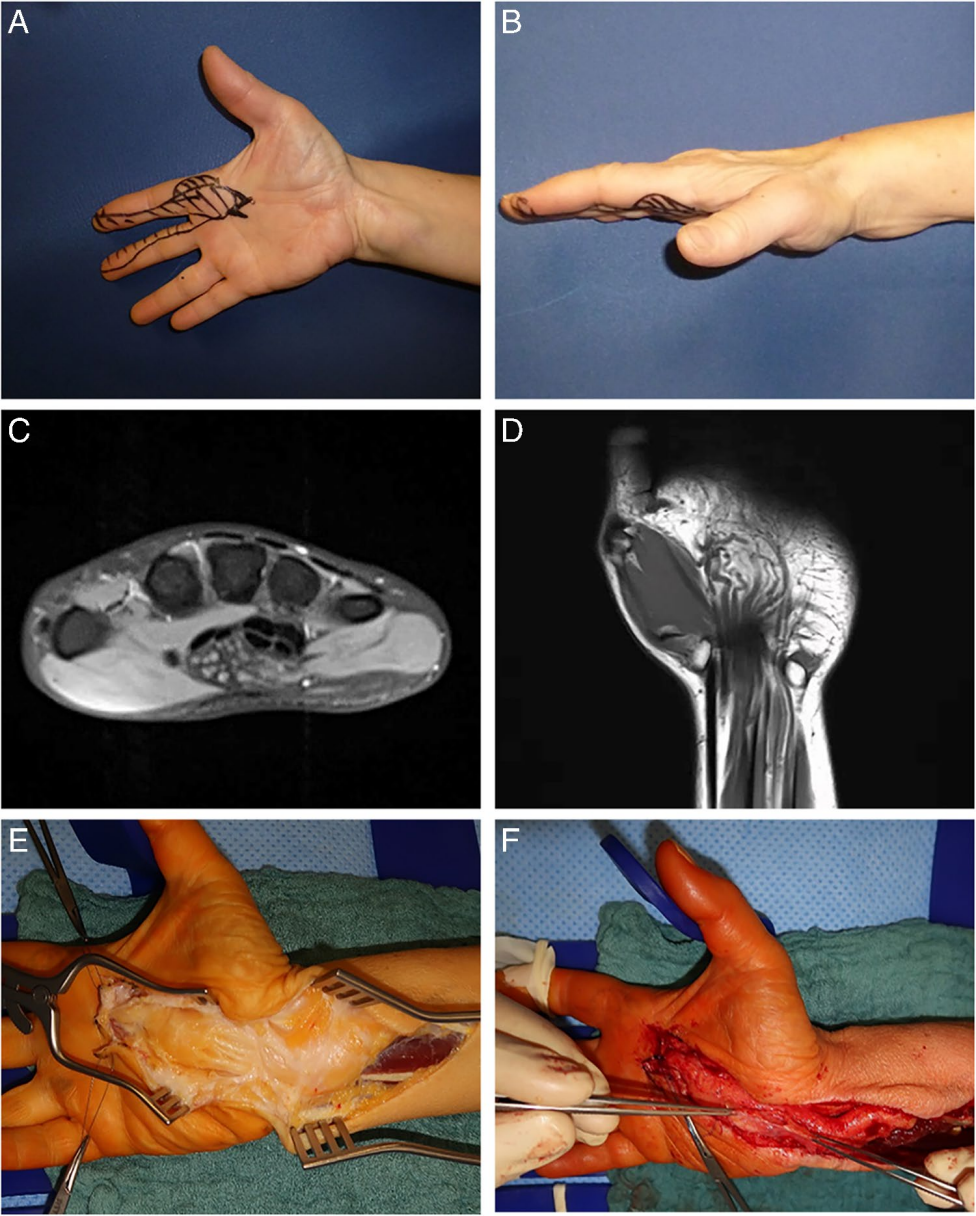
Clinical findings in patients with lipomatosis of the median nerve. **A**, **B** Clinical images showing a mass at the wrist. Areas with impairment of sensitivity are marked in black color. **C**, **D** MRI images revealing typical features of LPN of the median nerve with spaghetti-like nerve bundles. **E**, **F** Intraoperative findings of a giant median nerve at the wrist; the median nerve was covered with a fascial sheet after decompression

Clinical details were available for the four in-house patients. All four patients initially suffered from typical symptoms of carpal tunnel syndrome such as paresthesia or numbness of the thumb and index, and middle fingers. Clinical examination revealed positive Tinel’s sign and Phalen’s test. A palpable tumor mass at the wrist was present in three patients (Fig. 1). Neurophysiological study of the median nerve showed prolongation of the distal motor latency (DML) in all four patients. MRI-Imaging revealed a significant enlargement of the median nerve with typical features of LPN (fusiform nerve enlargement, fatty proliferation, coaxial-cable-like appearance on axial planes, and a spaghetti-like appearance [4]).

After noninvasive diagnostic workup, the patients underwent surgical treatment with an open release of the carpal tunnel and biopsy of the mass, either before (patient 2) or during surgical intervention. One patient presented intraoperatively with a constriction of the median nerve due to a thickened epineurium and perineurium. Therefore, neurolysis was performed and the median nerve was covered with a fascial sheet to prevent postoperative scarring. One patient (case 4) underwent initial decompression of the carpal tunnel in an external center without preoperative MRI-Imaging. Intraoperatively, a gigantic median nerve was found. After initial surgery, the patient reported improvement of the numbness of the thumb and index and middle fingers but was still suffering from paresthesia. The patient was referred to our center for further diagnostic workup. After MRI-imaging, the patient underwent surgical therapy at our center with neurolysis of the enlarged median nerve. Additionally, biopsies were taken for histopathological analysis. The two consultation cases were treated by unspecified surgical resection of the mass as noted from the submitted specimens.

### Follow-up, clinical outcome, and other associated diseases

Post-surgery, all patients reported initial improvement of symptoms. However, one patient reported persistent pain, edematous swelling of the right hand 4 weeks after surgery, and a complex regional pain syndrome (CRPS), which was treated by methylprednisolone, pregabalin, and vitamin C as well as DMSO/ambroxol cream, resulting into recovery.

One patient presented with recurrence of symptoms and a slowly growing palpable mass at the wrist 10 years after initial surgical decompression. Revision surgery revealed significant scarring of the former carpal ligament. Consequently, after decompression, the enlarged median nerve was secondarily covered with a fascial sheet to reduce the recurrence of scarring. Although details regarding other associated genetic disorders are not complete, bilateral LPN of the median nerve as possible feature of a genetic predisposition was observed in one patient (1/6 = 16.7%). The same patient presented with histologically verified extensive capillary-venous-type angiomatosis in the paravertebral soft tissue.

### Histopathological findings

**Fig. 2:**
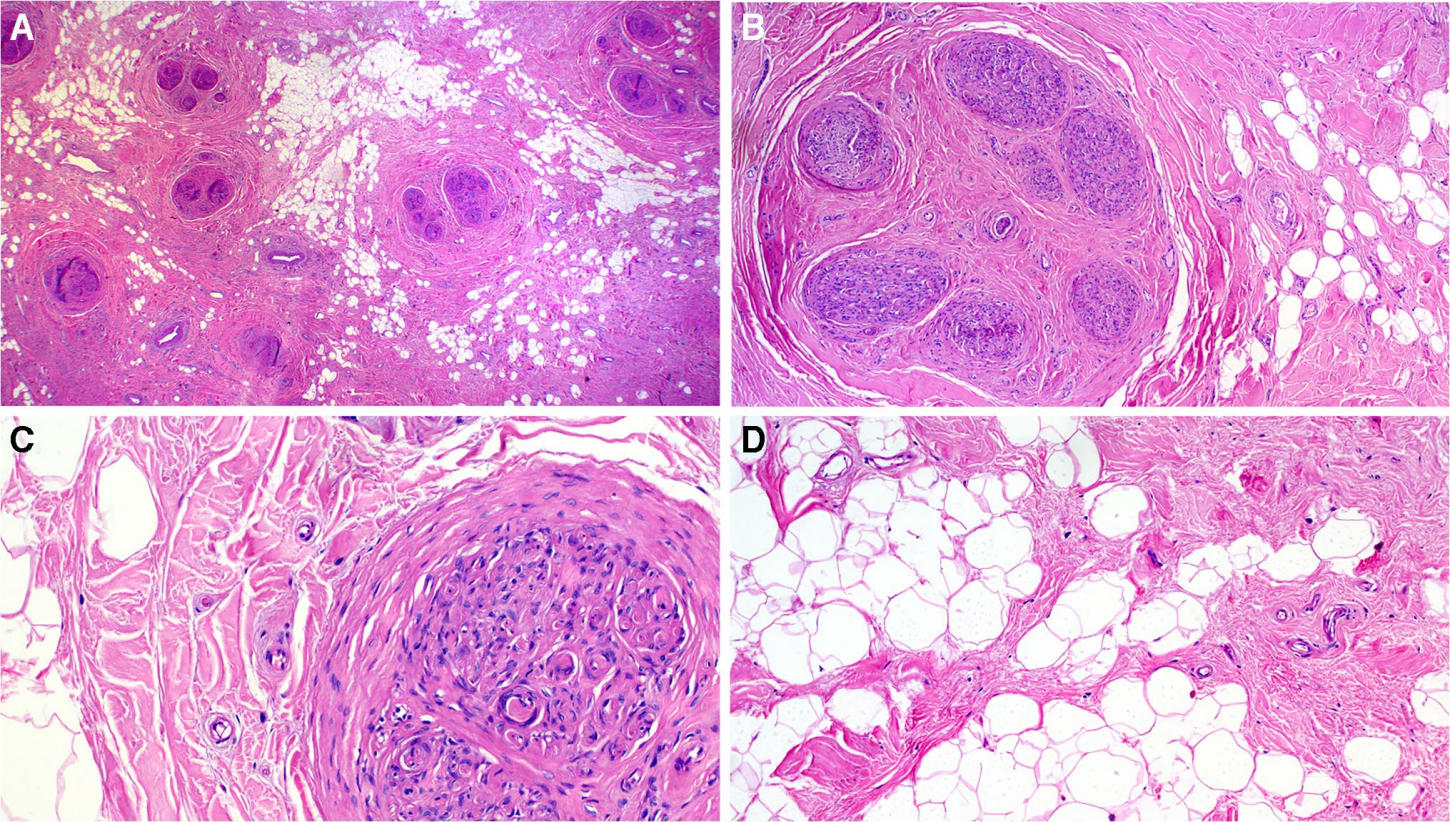
Representative examples of the histopathological findings in lipomatosis of the median nerve. **A** This resected lesion shows abnormal amount of fibrofatty tissue entrapping multiple nerve fascicles, note increased thick-walled vessels scattered in the background. **B** Another area showing the prominent endoneurial fibrosis involving one nerve fascicle. **C** Higher magnification of perineurial fibrosis. **D** Higher magnification of the fibrofatty tissue showing no atypia (this pattern represents the most frequent finding in small biopsies and is usually misinterpreted as nondiagnostic)

Histologically, all cases were similar, being composed of an abnormal amount of normal-looking mature fatty tissue containing variable amount of disordered fibrous tissue forming linear bands and small aggregates. Based on the type of biopsy and tissue amount, variable numbers of small or thick nerve fascicles were seen entrapped within the fibrolipomatous background, associated with a variable degree of perineurial and endoneurial fibrosis (Fig. 2). The resected paravertebral soft tissue mass in case 6 revealed diffuse arrangement of medium-sized venous and capillary-type vessels without atypia and without associated increase in fatty tissue, consistent with angiomatosis (not shown). Notably, three cases received a merely descriptive reporting without mentioning LPN in the pathology report.

### Molecular findings

Genetic analysis using snapshot assay constructed to detect hotspots mutations in *PIK3CA* revealed an identical pathogenic *PIK3CA* mutation (p.H1047R; c.3140A>G) in 5 of 6 cases (83.3%). In one patient, we tried separate manual microdissection of the nerves and the hamartomatous tissue; we detected the same mutation in both samples. However, due to the manual nature of microdissection, we cannot verify the presence of the mutation in the nerves with certainty as contamination cannot be reliably ruled out. Molecular testing of the angiomatosis lesion of soft tissue in case 2 failed probably due to the decalcification of the resection specimen. No clinicopathological differences were observed regarding the single *PIK3CA* wild-type case compared to the mutated cases.

## Discussion

Lipomatosis of peripheral nerves (LPN) is a distinctive entity first described by Silverman and Enzinger in 1985 as fibrolipomatous hamartoma of peripheral nerves [5]. Although any peripheral nerve can be affected, most cases involve the median nerve [1]. The lesion is composed of fibroadipose tissue, expanding and replacing the epineurium and encasing and separating nerve bundles, associated with variable perineurial fibrosis. Therefore, LPN cannot be resected entirely without causing damage of the affected peripheral nerve. Since LPN essentially recapitulates the normal tissue, a hamartomatous origin has been postulated. However, the widely used original name “fibrolipomatous hamartoma of peripheral nerve” has been replaced by the terminology “lipomatosis of nerves” in the current WHO classification. LPN is generally a slowly growing lesion. Patient’s age at time of diagnosis varies greatly. In many cases, the lesion is diagnosed soon after birth or is congenital, while cases diagnosed as late as the fifth or sixth decade of life have also been reported [1]. LPN usually presents as unilateral lesion, but bilateral cases have also been documented [6]. Associations with macrodactyly (macrodystrophia lipomatosa) have been reported frequently, with prevalence of up to one third of all cases [7]. However, in our analysis, we could not find a single patient with LPN associated with macrodactyly.

The clinical diagnosis of LPN can be challenging. If there is no obvious mass lesion, symptoms such as numbness or pain can be nonspecific, as they can also be associated with trauma, other neural tumor entities, or just a result of overtraining [8–10]. Certain imaging criteria are helpful in diagnosis of LPN. MRI, especially in t1 sequences, shows hypointense “cable wire”-like nerve bundles surrounded by hyperintense adipose tissue [11]. However, diagnosis should be verified by histopathological analysis of biopsies to rule out malignancies, particularly lipomatous neoplasms [12].

Although morphologically being a simple bland lesion, histopathological diagnosis of LPN is often a challenge due to two major reasons: 1) the entity is very rare; hence, majority of pathologists (except for soft tissue pathologists) might not be aware of it and 2) the histological biopsy findings are nonspecific and practically indistinguishable from normal tissue, resulting frequently in descriptive diagnoses of “normal fibrofatty tissue or negative for pathological findings or neoplasms. This is further enhanced by the fact that only small tissue fragments are usually submitted in an attempt to avoid scarifying the nerves, so the mass-forming appearance of the lesion is only rarely seen by pathologists [5]. In line with this, three of our six cases received initially a merely descriptive histopathology reporting (only those cases seen by a soft tissue pathologist were reported as LPN). Clinically, LPN of the median nerve can be distinguished easily from potential differential diagnoses such as schwannoma, neurofibroma, and giant cell tumor by its characteristic imaging findings [13].

Recent exome sequencing studies reported somatic activating *PIK3CA* gene mutations in LPN with or without associated macrodactyly/overgrowth, leading to an establishment of the concept of *PIK3CA*-related “overgrowth syndromes” [14, 15]. Our results confirm these recent studies and show the presence of *PIK3CA* mutations in the majority of cases, independent of absence of macrodactyly. Accordingly, we advocate the use of *PIK3CA* mutation testing as a valuable surrogate for confirming diagnosis in equivocal cases on limited biopsies. Clustering of the mutations at codon 3410 points to the reliable rational use of hotspots testing methods to recognize most of the mutations expected in these lesions.

The role of *PIK3CA* mutations in the pathogenesis of diverse localized or diffuse overgrowth syndromes has been increasingly recognized. One of our cases presented with bilateral LPN involving the median nerve in addition to soft tissue angiomatosis of the paravertebral region, suggesting either an inherited or a mosaic disorder, in line with the recently reported *PIK3CA*-related vascular anomalies [16]. Notably, the reported *PIK3CA*-associated vascular lesions were clinically isolated and were not associated with other disorders or with LPN as in one of our cases. Moreover, *PIK3CA* mutations were also reported in more extensive phenotypes in the disorders collectively termed *PIK3CA*-related “overgrowth spectrum=PROS” including Klippel–Trenaunay syndrome [17]. In one previous study, entire limb overgrowth was reported in two patients with LPN and *PIK3CA*, but no case had reported vascular anomalies. Accordingly, our case 2 represents a novel addition to the clinicopathological associations of LPN.

Surgical treatment of LPN of the median nerve should be initiated individually. Our approaches vary depending on the symptoms, radiologic, and clinical as well as on intraoperative findings. A conservative treatment regimen is possible if the patient does not suffer from pain or neurological impairment. In all other cases, a surgical procedure should be indicated to resolve the symptoms and to avoid permanent nerve damage. The first step of the surgical treatment is an open carpal tunnel decompression. Moreover, taking biopsies is recommended to rule out malignancies. A resection of the mass is impossible without causing permanent damage to the affected nerve and should not be performed.

In some cases, as described in the first patient, no further surgical therapy is necessary (if there are no other pathological findings). However, the third case shows that if the surgeon encounters a constriction of the median nerve due to a thickened epineurium and perineurium, we suggest performing a neurolysis. Additionally, the nerve can be covered with a fascial sheet to prevent scarring and improve gliding [15, 16]. Another indication for neurolysis and coverage of the median nerve with a fascial sheet is postoperative scarring, as highlighted in patient 2. Tumor debulking is considered another surgical option. Nevertheless, this rather invasive procedure puts the median nerve in danger for injury and leads to an intense healing response with increased scarring. Therefore, we recommend it only for distinct cases [17].

## Conclusion

Lipomatosis of the median nerve is a very rare cause of carpal tunnel syndrome. Early surgical decompression of the carpal tunnel as well as biopsies of the mass is mandatory to achieve improvement of symptoms and to avoid long-term damage of the nerve as well as to rule out malignancies. Recurrent *PIK3CA* mutations in these rare but diagnostically and therapeutically challenging lesions represent a valuable surrogate molecular marker for confirming diagnosis in limited biopsies or clinically ambiguous cases. Moreover, the role of these mutations as a potential therapeutic target using mTOR inhibitors like Everolimus and other substances remains to be verified in future studies.

## Data Availability

The datasets generated during and/or analyzed during the current study are not publicly available due to legal constrictions but are available from the corresponding author upon reasonable request.

## References

[1] Tahiri Y (2013). *Lipofibromatous hamartoma of the median nerve: a comprehensive review and systematic approach to evaluation, diagnosis, and treatment*. J Hand Surg Am.

[2] Marek T (2019). *Strengthening the association of lipomatosis of nerve and nerve-territory overgrowth: a systematic review*. J Neurosurg.

[3] Lurkin I (2010). *Two multiplex assays that simultaneously identify 22 possible mutation sites in the KRAS, BRAF, NRAS and PIK3CA genes*. PLoS One.

[4] Marom EM, Helms CA (1999). *Fibrolipomatous hamartoma: pathognomonic on MR imaging*. Skeletal Radiol.

[5] Silverman TA, Enzinger FM (1985). *Fibrolipomatous hamartoma of nerve. A clinicopathologic analysis of 26 cases*. Am J Surg Pathol.

[6] Chandler EM, Chen CM, Spector JA (2009). *Bilateral fibrolipoma of the median nerve*. J Plast Reconstr Aesthet Surg.

[7] Azeemuddin M (2018). *Fibrolipomatous hamartoma of the median nerve with macrodystrophia lipomatosa*. Cureus.

[8] Rosenauer R (2020). *Complications after operatively treated distal radius fractures*. Arch Orthop Trauma Surg.

[9] Weschenfelder W (2019). *Acute atraumatic carpal tunnel syndrome due to flexor tendon rupture following palmar plate osteosynthesis in a patient taking rivaroxaban*. Arch Orthop Trauma Surg.

[10] Woertler K (2010). *Tumors and tumor-like lesions of peripheral nerves*. Semin Musculoskelet Radiol.

[11] Marek T (2021). *What's known and what's new in adipose lesions of peripheral nerves?*. Acta Neurochir (Wien).

[12] Toft F (2022). *Surgical resection of a giant intramuscular lipoma of the biceps brachii: a case report and review of the literature*. Arch Orthop Trauma Surg.

[13] Razzaghi A, Anastakis DJ (2005). *Lipofibromatous hamartoma: review of early diagnosis and treatment*. Can J Surg.

[14] Blackburn PR (2020). *PIK3CA mutations in lipomatosis of nerve with or without nerve territory overgrowth*. Mod Pathol.

[15] Rios JJ (2013). *Somatic gain-of-function mutations in PIK3CA in patients with macrodactyly*. Hum Mol Genet.

[16] Boccara O (2020). *Soft tissue angiomatosis: another PIK3CA-related disorder*. Histopathology.

[17] Yeung KS (2017). *Somatic PIK3CA mutations in seven patients with PIK3CA-related overgrowth spectrum*. Am J Med Genet A.

